# PCP-ML: Protein characterization package for machine learning

**DOI:** 10.1186/1756-0500-7-810

**Published:** 2014-11-18

**Authors:** Jesse Eickholt, Zheng Wang

**Affiliations:** Department of Computer Science, Central Michigan University, Mount Pleasant, MI 48859 USA; School of Computing, University of Southern Mississippi, Hattiesburg, MS 39406 USA

**Keywords:** Protein structure prediction, Protein characterization, Protein software package, Machine learning

## Abstract

**Background:**

Machine Learning (ML) has a number of demonstrated applications in protein prediction tasks such as protein structure prediction. To speed further development of machine learning based tools and their release to the community, we have developed a package which characterizes several aspects of a protein commonly used for protein prediction tasks with machine learning.

**Findings:**

A number of software libraries and modules exist for handling protein related data. The package we present in this work, PCP-ML, is unique in its small footprint and emphasis on machine learning. Its primary focus is on characterizing various aspects of a protein through sets of numerical data. The generated data can then be used with machine learning tools and/or techniques. PCP-ML is very flexible in how the generated data is formatted and as a result is compatible with a variety of existing machine learning packages. Given its small size, it can be directly packaged and distributed with community developed tools for protein prediction tasks.

**Conclusions:**

Source code and example programs are available under a BSD license at http://mlid.cps.cmich.edu/eickh1jl/tools/PCPML/. The package is implemented in C++ and accessible as a Python module.

**Electronic supplementary material:**

The online version of this article (doi:10.1186/1756-0500-7-810) contains supplementary material, which is available to authorized users.

## Findings

Machine Learning (ML) techniques have been successfully applied to a variety of protein related classification tasks. In particular, machine learning has proven quite useful in the area of protein structure prediction and resulted in the development of a number of tools and particular applications. These include the prediction of a protein’s secondary structure [1, 2], residue solvent accessibility [1], residue-residue contacts and contact maps [3, 4], residue order/disorder [5, 6], fold recognition [7] and protein model quality [8]. These tools, while useful in their own right, also form part of larger protein structure tools and tertiary structure prediction pipelines (e.g., MULTICOM [9] and I-TASSER [10]).

In general, machine learning methods work on a feature space which characterizes an object or event. The machine learning methods attempt to learn some meaningful relation between elements in the feature space and/or a map between the feature space and classifications. For most protein prediction tasks, the primary feature space is the protein’s sequence and/or data directly derived thereof (e.g., sequence profile). As machine learning techniques are mathematical models, the sequence data (e.g., FASTA files, multiple sequence alignments, etc.) must be read in and then converted to a meaningful numerical format.

**Figure 1:**
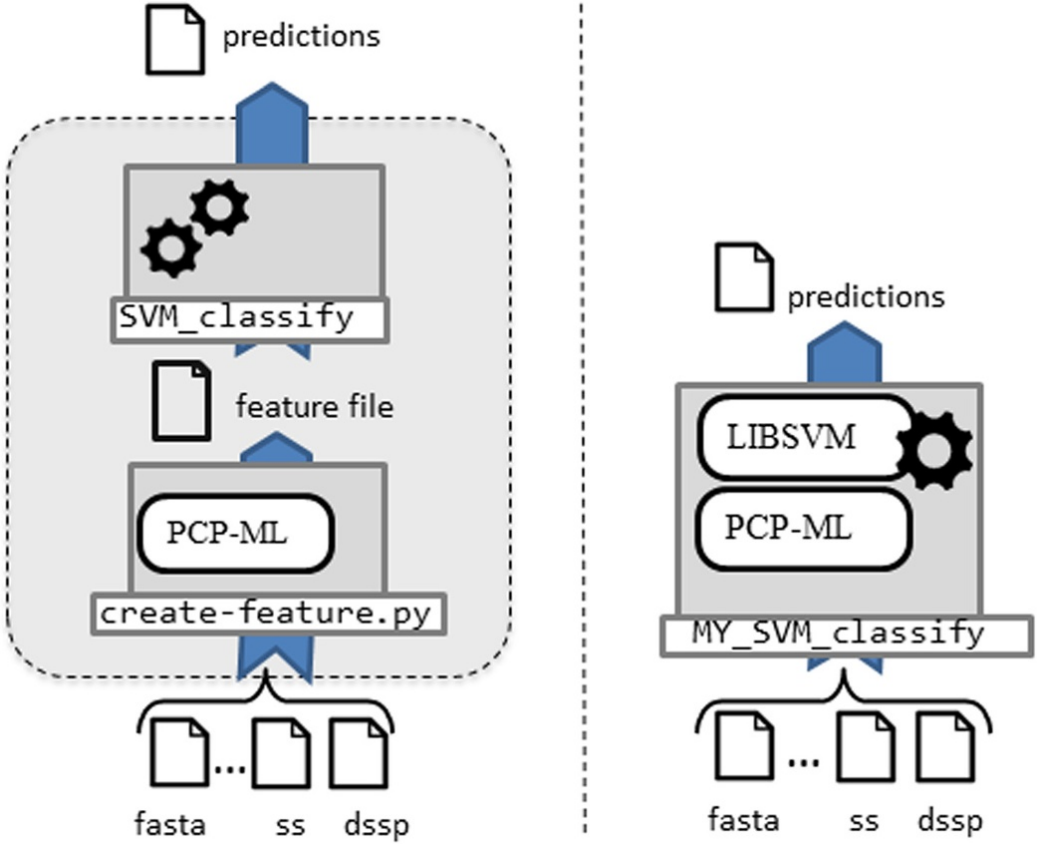
**PCP-ML can be used to create a quick, stand-alone feature generation program or used in conjunction with other libraries.** The left side of the figure illustrates how PCP-ML can be used to create feature files which are then fed into existing ML tools (e.g., SVM^light^) to generate a final prediction. In this case, PCP-ML is used to create a feature file that becomes the input to an existing machine learning tool, and this tool makes the final prediction. The right side of the figure shows PCP-ML packaged with other machine learning libraries for a complete, custom solution for protein prediction tasks. In this case, one program will read the input and generate the predictions. The round boxes represent libraries or packages and the labelled boxes represent programs or scripts.

Here we present PCP-ML, a package of methods that characterize a protein for machine learning tasks. The package can be of use in any protein prediction problem in which the input is the protein’s primary sequence. We have tailored PCP-ML to protein structure prediction tasks in particular. Our package was inspired by existing protein sequence libraries such as in Bio++ [11], Biopython [12] and SeqAn [13], but differs in its focus on machine learning, compact size and additional functionality for protein structure prediction. It provides a stable, expandable and lightweight set of methods that can be used when developing machine learning based tools in structural bioinformatics and to the best of our knowledge it is the first library or package of its type. The package is written in C++ but accessible as a Python module. This allows for rapid prototyping in a scripting language. Yet due to the scope and size of PCP-ML, it is much more amenable to being embedded as a part of an application or tool than many existing libraries. The primary purpose of our software package is to provide a concise, tested set of functions that can be used to generate feature files for existing machine learning tools (such as SVM^light^ [14] or NNrank [15]) or as a built in component for a stand-alone protein structure prediction tool. Note that the PCP-ML package itself does not provide any functionality to train prediction tools but rather it focuses on the pre-processing and data access phase, converting protein sequence data into a format that can be used with machine learning. Figure 1 illustrates how PCP-ML could be incorporated into a prediction pipeline, either as a built-in component or as a stand-alone feature generation program feeding into an off-the-shelf machine learning toolkit.

### Methods

**Figure 2:**
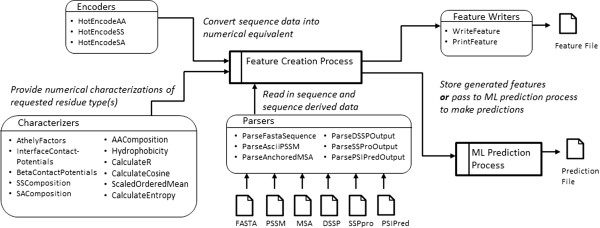
**Dataflow diagram for common uses of PCP-ML.** The feature generation process accesses sequence data through the Parsers. Based on the prediction task, numerical data that characterizes a protein’s sequence is provided through the Characterizers and Encoders. The generated features are then saved to a file or passed to a machine learning prediction process to make an end prediction.

The design and functionality of PCP-ML is based on our experience with machine learning in protein structure prediction tasks as well as a survey of methods documented in the literature. We have broken PCP-ML into primarily three components: Parsers, Characterizers and Encoders, and Feature Writers/Generators. Table 1 summarizes the majority of the methods available in each component and Figure 2 depicts how data flows between the Parsers, Characterizers, Encoders and Feature Writers. To see how the components are used in practice, see Additional file 1 which contains some sample scenarios for using PCP-ML (in both C++ and Python).

**Table 1:** **Major methods provided by each component of PCP-ML**

Parsers and encoders	Characterizers	Feature writers and generators
ParseFastaSequences	AthelyFactors	PrintFeatures
ParseSSProOutput	InterfaceContactPotentials	WriteFeatures
ParsePSIPredOutput	BetaContactPotentials	
ParseAsciiPSSM	SSComposition	
ParseAnchoredMSA	SAComposition	
ParseDSSPOutput	AAComposition	
HotEncodeAA	Hydrophobicity	
HotEncodeSS	CalculateR	
HotEncodeSA	CalculateCosine	
	ScaledOrderedMean	
	CalculateEntropy

### Parsers

As almost all protein structure prediction tasks start with the protein’s sequence and sequence profile, PCP-ML provides several methods to parse FASTA files, anchored multiple sequence alignments (MSAs), output from DSSP [16], and position specific scoring matrices (PSSMs) from PSI-BLAST [17]. Many higher level prediction tasks also make use of predicted secondary structure and predicted solvent accessibility. Therefore, we have included parsers for common output formats for these types of predictions. In particular, PCP-ML can read files generated from SSPro [1] and PSIPRED [2].

Here, we note that we do not include a parser for PDB files (i.e., a common format used by the Protien Data Bank [18] for protein structure). Our rationale for not including a PDB parser is that for most prediction tasks, the structural information that would be contained in a PDB file is not available and hence a PDB parser is not needed for the production of end-user protein structure prediction tools. Interested readers may find a PDB parser included with Biopython [12] or ESBTL [19].

### Characterizers and encoders

The input into machine learning methods is numerical and as a result it is necessary to encode data such as secondary structure (SS), solvent accessibility (SA) and amino acid (AA) type. One approach to this end is to convert each SS, SA and AA type to vectors of length 3, 2 and 20, respectively. In each vector, all of the values are 0 except for one value which depending on its position in the vector signifies the type (e.g. 100 represents a helix while 001 encodes for a coil). This type of encoding is often referred to as hot encoding, or orthogonal encoding [20], and allows for a numerical conversion without arbitrarily imposing an ordering on the encoding. PCP-ML contains three methods for hot encoding.

There are a number of ways to characterize a protein’s sequence and the PCP-ML package includes many of these. Perhaps the most obvious is to represent amino acid residues by numerical values stemming from statistical studies on experimentally determined structures. Included in PCP-ML are pair-wise contact potentials [21], beta sheet pairing potentials [22], and hydrophobicity [23]. We also included the Atchley factors for each amino acid [24]. These factors represent each amino acid type in a five dimensional space in which similar amino acids are grouped together and the proximity of any two amino acids is a measure of their similarity.

Proteins can also be characterized by their content. PCP-ML contains methods which calculate the percent content of a protein by secondary structure type (i.e., helix, beta sheet, or coil/loop), solvent accessibility (i.e., buried or exposed) or amino acid residue type. This information is a way to characterize a protein globally (i.e., irrespective of residue index). This approach can also be applied at the residue level using an anchored MSA or PSSM. Using either an MSA or PSSM, it is possible to calculate the relative frequency of each type of AA at a position in the sequence as well as the amount of information contained at that position.

Finally, a protein can be characterized by patterns or correlations in sequence data. Thus, we have included in PCP-ML methods to calculate the information contained in a vector or the correlation or similarity between two vectors. We also mention here that most methods have the option of returning scaled values such that the feature values are between 0 and 1. This is required by some machine learning methods. Table 2 provides a brief summary of the functionality provided by each characterizer in PCP-ML.

**Table 2:** **Description of each Characterizer contained in PCP-ML**

Name of characterizer	Brief description of functionality provided
AtchleyFactors	Characterizes five major aspects of an amino acid with real number values. The values were obtained via a statistical analysis of amino acids when looking at polarity, secondary structure, molecular size , amino acid composition and charge. These values were reported in [24].
InterfaceContactPotentials	Characterizes contact potential between two residues. These contact potentials come from a statistical analysis performed on contacts in protein interfaces. They were reported in [18].
BetaContactPotentials	Characterizes the contact potential for two residues in two beta sheets. These values come from a study of contact potentials of residues in cross strand pairings in beta sheets. They were reported in [22].
SSComposition	Determine the percentage of each secondary structure (SS) type in a string representing the secondary structure of the entire protein.
SAComposition	Determine the percentage of solvent accessibility from a string representing the solvent accessibility of the entire protein.
AAComposition	Determine the percentage of each amino acid in a protein sequence.
Hydrophobicity	Characterizes the hydrophobicity of a residue. These values come from a study on hydrophobicity and helical propensity in [23].
CalculateR	Calculates the Pearson correlation coefficient for the elements of two feature vectors.
CalculateCosine	Calculates the cosine between two feature vectors.
ScaledOrderedMean	Calculates the nth ordered mean for the Amino Acid, Secondary Structure or Solvent Accessibility string.
CalculateEntropy	Calculates the Shannon entropy for a vector of probabilities

### Feature generators/writers

The input format for standard machine learning packages (e.g., SVM^light^, NNrank, etc.) varies but typically consists of a text file in which each line represents a training or classification example. Some packages require the features to be numbered as well. PCP-ML provides feature writers which can print out features (optionally with number) and/or save them to a file. This functionality allows users to use PCP-ML to create stand-alone feature generation programs that they can package with standard machine learning programs or tie feature generation directly into their tools. Note that it is difficult to accommodate file formats for all machine learning packages. The feature writers we developed and included allow a user to print the features along with feature numbers and/or the labels/targets themselves. The targets and feature numbers can be easily modified via the parameters passed to the feature writing functions.

### Conclusions

PCP-ML is a software package to characterize proteins for machine learning applications in protein structure prediction as well as more general protein related prediction tasks. It provides a number of functions that allow for rapid prototyping and testing of methods and easy deployment of developed tools. The package can be used to create feature generation programs compatible with popular machine learning tools or compiled into stand-alone applications. As an open source project, it is freely available to the community and can be modified and extended as needed.

## Availability and requirements

Project name: PCP-ML

Project home page: http://mlid.cps.cmich.edu/eickh1jl/tools/PCPML/

Operating System(s): Linux, Mac OS X

Programming Language: C++, Python

Other requirements: C++ compiler

License: BSD

Any restrictions to use by non-academics: None

PCP-ML is written in C++ and available in both source code and a Python module. These are available at http://mlid.cps.cmich.edu/eickh1jl/tools/PCPML/. At the site, users can also find examples, a tutorial, access additional documentation and learn about porting the package to other languages such as Perl or Octave.

## Electronic supplementary material

Additional file 1: **A stand-alone webpage with an example use of PCP-ML.** (ZIP 5 KB)
