# Supplementary material for: PCP-ML: Protein characterization package for machine learning
Source: BMC Res Notes. 2014 Nov 18;7:810. doi: 10.1186/1756-0500-7-810 (PMC4246511; doi:10.1186/1756-0500-7-810)
Supplement: Supplementary file 1 — Additional file 1: A stand-alone webpage with an example use of PCP-ML. (ZIP 5 KB) [file 13104_2014_3335_MOESM1_ESM.zip › index.html]

PCP-ML - Example


# PCP-ML

## Protein Characterization Package for Machine Learning

# Tutorial - C++

In this short tutorial, we will construct a feature generation script that could be used to train a protein residue order/disorder predictor. The files needed can be found in the tutorial directory in the PCP-ML code tarball.

### Reading in Data

For almost any use of ML, it is necessary to create, transform or encode your data into a form compatible with the ML framework. Typically, this means representing the data numerically and when working with protein data it has to be encoded as well. First, we will need to read in the raw data. The following snippet of code demonstrates the general procedure. The ReadFile method can be used to open a text file and grab the contents. Then a parser (e.g., ParseSSProOutput, ParseAsciiPSSM, etc) can be used to parse out the desired content. For non-standard input files, one can handle the data as they see fit. In this snippet, the *od* file is not a standard file format but one we created. It contains two lines with the first being the sequence and the second the true ordered or disordered state.

The following snip of code illustrates the libraries and headers that one will typically need to pull in. It also reads in and parses out the four input files.

```
#include <string>
#include <vector>
#include "FastaSequence.h"
#include "Utilities.h"
#include "Parsers.h"
 
using namespace PCP;
 
int main(int argc, const char *argv[]) {
 
  if(argc != 5) {
    Fail("Usage: generate-OD-feats <fasta> <sspro> <pssm> <od>");
  }
  std::string fasta_filename = argv[1];
  std::string sspro_filename = argv[2];
  std::string pssm_filename = argv[3];
  std::string od_filename = argv[4];
 
  // Read in sequence, check that one was read
  std::vector<std::string> lines;
  std::vector<FastaSequence> sequences;
  int sequence_count = 0;
  ReadFile(fasta_filename, lines);
  sequence_count = ParseFastaSequences(lines, sequences);
  if(sequence_count == 0) {
    Fail("No sequence was read from file " + fasta_filename);
  }
  FastaSequence primary_sequence = sequences[0];
 
  // Read in and parse SSpro (ie, secondary structure)
  lines.clear();
  std::string ss_seq;
  std::string ss;
  std::string sa;
  ReadFile(sspro_filename, lines); 
  ParseSSProOutput(lines, ss_seq, ss, sa);  
 
  // Read in and parse pssm
  lines.clear();
  std::vector< std::vector<double> > pssm;
  std::vector<double> information;
  ReadFile(pssm_filename, lines);
  ParseAsciiPSSM(lines, pssm, information, true);
 
  // Read in od, this is not a standard file so there
  // is not a parse, but we can copy out the lines needed.
  lines.clear();
  std::string od_seq;
  std::string od;
  ReadFile(od_filename, lines);
  od_seq = lines[0];
  od = lines[1];
 
  // GENERATE FEATURES HERE!!! 
 
  return 0;
}
```

### Encoding Features

In this particular feature generation program, we will run a small sliding window accross the sequence and encode some data for each residue in the sliding window. This approach is typical for protein structure prediction tasks such as this one. Since we are going to use some of the built-in encoders, we need to include the "Encoders.h" header file. Then we will use several encoders to add features to the feature vector. At the end, we print out the class along with all of the features.

```
  // Generate features for a sliding window of 9 residues
  // running from the 5th residue to length-5
  std::vector<double> features;
 
  for(unsigned int center = 4; center < sequences[0].Length() - 5; ++center) {
    features.clear();
 
    // For each residue in the window, push features on end of 
    // Feature vector
    for(unsigned int index = center - 4; index <= center + 4; ++index) {
      // Add sequence information
      HotEncodeAA(primary_sequence[index], features);
      // Add likelihoods for residue types at this position
      features.insert(features.end(), pssm[index].begin(), pssm[index].end());
      // Add Secondary Structure information
      HotEncodeSA(sa[index], features);
      HotEncodeSS(ss[index], features);
    }
 
    // Write out target
    if(od[center] == 'O') {
      std::cout << "-1 ";
    } else {
      std::cout << "+1 ";
    }
 
    // Follow up by writing out features
    PrintFeatures(features, true, 1);
 
  }
```

The last statement prints out the features for the residue along with a feature number that starts at 1. This output is compatible with the feature file format of SVMlight. We could have also saved the features to a file using the WriteFeatures method.

### Compiling and Running

Included in the tutorials directory is a simple Makefile that compile the feature generation program. Make sure that you compile the library first (i.e., run the main Makefile for the library). The executable can be tested using four files supplied for the hypothetical protien ProtZ.  

```
 ./generate-OD-feats ProtZ.fasta ProtZ.ss_sa ProtZ.pssm ProtZ.od
```

  
This should generate several lines of feature data, each starting with a class and containing numbered features. The following is typical of the output you will obtain...

```
+1 1:0.00000 2:0.00000 3:0.00000 4:0.00000 5:0.00000 6:0.00000 7:0.00000 8:0.00000 9:0.00000 10:0.00000 11:0.00000 12:0.00000 13:1.00000 
14:0.00000 15:0.00000 16:0.00000 17:0.00000 18:0.00000 19:0.00000 20:0.00000 21:0.73106 22:0.50000 23:0.11920 24:0.11920 25:0.04743 
26:0.26894 27:0.11920 28:0.11920 29:0.04743 30:0.01799 31:0.01799 32:0.26894 33:0.04743 34:0.01799 35:0.99753 36:0.88080 37:0.50000 
38:0.01799 39:0.01799 40:0.04743 41:0.00000 42:1.00000 43:0.00000 44:0.00000 45:0.00000 46:0.00000 47:0.00000 48:0.00000 49:0.00000 
50:0.00000 51:0.00000 52:0.00000 53:0.00000 54:0.00000 55:0.00000 56:0.00000 57:0.00000 ...
```

# Tutorial - Python

It is also possible to create feature generate scripts with Python (version 2). The functionality of PCP-ML remains largely the same between the C++ and Python versions. There are a few differences which will be discussed below. First, we need to build the Python module.

### Building the Python Module

First, make sure you compile the base code (i.e., run the main Makefile) and then run the Makefile in the python\_bindings subdirectory to create the Python specific library and module. If you wish, you can move the \_PCPML.so, PCPML.py and PCPML\_Utilities.py files to a location that is in your library path for Python.

### Using the PCP-ML Module

In is necessary to import both the PCPML module and the PCPML\_Utililties module. The later gives you access to the ReadFile function. Because the methods provided in PCP-ML use elements from the C++ Standard Template Library, it is slightly more cumbersome to access them through Python. Note that to parse a PSSM, a matrix and vector of doubles must first be created and then passed to ParseAsciiPSSM. To parse secondary structure and solvent accessiblity, we use wrapper functions that take a RETURN\_TYPE parameter and return a string with the requested information (e.g., sequence, secondary structure or solvent accessiblity).

```
import sys
 
# Make sure that you have built the Python bindings first
# and set the path
#sys.path.append('PATH_TO_COMPILED_BINDINGS_HERE');
sys.path.append('/home/eickholtj/sandbox/PCP-ML/python_bindings');
 
import PCPML
from PCPML_Utilities import ReadFile
 
if(len(sys.argv) != 5):
    print "Usage:", sys.argv[0], "<fasta> <sspro> <pssm> <od>";
    sys.exit(-1);
 
fasta_filename = sys.argv[1];
sspro_filename = sys.argv[2];
pssm_filename = sys.argv[3];
od_filename = sys.argv[4];
 
lines = PCPML.Lines();
 
# Read in sequence
ReadFile(fasta_filename, lines);
header = lines[0];
sequence = "";
for i in range(1, lines.size()):
    sequence = sequence + lines[i]
 
# Read in ss sa
lines.clear();
ReadFile(sspro_filename, lines);
ss_seq = PCPML.ParseSSProOutput(lines, PCPML.SEQUENCE);
ss = PCPML.ParseSSProOutput(lines, PCPML.SS);
sa = PCPML.ParseSSProOutput(lines, PCPML.SA);
 
# Read in pssm
lines.clear();
ReadFile(pssm_filename, lines);
pssm = PCPML.Matrix();
inf = PCPML.VectDouble();
PCPML.ParseAsciiPSSM(lines, pssm, inf, 1);
 
# Read in od
lines.clear();
ReadFile(od_filename, lines);
od_seq = lines[0];
od = lines[1];
 
f = PCPML.VectDouble();
for center in range(4, len(sequence)-6):
    f.clear();
 
    for idx in range(center-4, center+5):
        PCPML.HotEncodeAA(sequence[idx], f);
        for i in range(0,pssm[i].size()):
            f.push_back(pssm[idx][i]);
 
 
    # Write out features 
    if od[center] == 'D':
        sys.stdout.write("1 ");
    else:
        sys.stdout.write("-1 ");
    PCPML.PrintFeatures(f, 1, 1);
```

### Running

Invoke from the command line.   

```
python2 generate-OD-feats.py ProtZ.fasta ProtZ.ss_sa ProtZ.pssm ProtZ.od
```
